# Cumulative occupational exposure to inorganic dust and fumes and invasive pneumococcal disease with pneumonia

**DOI:** 10.1007/s00420-022-01848-6

**Published:** 2022-03-09

**Authors:** Kjell Torén, Paul D. Blanc, Rajen Naidoo, Nicola Murgia, Leo Stockfelt, Linus Schiöler

**Affiliations:** 1 Occupational and Environmental Medicine, School of Public Health and Community Medicine, Institute of Medicine, Sahlgrenska Academy, University of Gothenburg, Box 414, 405 30 Gothenburg, Sweden; 2 Discipline of Occupational and Environmental Health, University of KwaZulu-Natal, Durban, South Africa; 3 Division of Occupational and Environmental Medicine, Department of Medicine, University of California, San Francisco, CA USA; 4 Section of Occupational Medicine, Respiratory Diseases and Toxicology, University of Perugia, Perugia, Italy; 5 Department of Occupational and Environmental Medicine, Sahlgrenska University Hospital, Gothenburg, Sweden

**Keywords:** Pneumonia, Pneumococci, Inorganic dust, Occupational exposure, Case, Control

## Abstract

**Purpose:**

Occupational exposure to inorganic dust and fumes in the year preceding disease has been associated with increased pneumococcal pneumonia risk, but the impact of prior cumulative exposure has not been characterized.

**Methods:**

We studied 3184 cases of invasive pneumococcal disease with pneumonia. The case index date was the day the infection was diagnosed. We selected six controls for each case from the Swedish population registry; each control was assigned the index date of their corresponding case. We linked job histories to a job-exposure matrix to calculate a cumulative exposure index, intensity-years, by multiplying the duration (maximum 5 years) of each exposure with the level of exposure (0 for unexposed, 1 for low and 4 for high). We used conditional logistic analyses to estimate the odds ratio (OR) of invasive pneumococcal disease with pneumonia adjusted for comorbidities, educational level, income and other occupational exposures.

**Results:**

Taking other occupational exposures into account, greater than 5 intensity-years of exposure to silica dust or to fumes was each associated with increased odds for invasive pneumococcal disease with pneumonia (OR 2.53, 95% CI 1.49–4.32) and (OR 2.24, 95% CI 1.41–3.55), respectively. Five intensity-years or less of exposure to silica dust or fumes manifested lower odds (OR 1.45, 95% CI 1.20–1.76) and (OR 1.05, 95% CI 0.94–1.16), respectively.

**Conclusion:**

This study adds evidence that the risk of pneumococcal pneumonia increases with increasing cumulative exposure to dust and fumes, indicating the importance of cumulative exposure.

## Introduction

*Streptococcus pneumoniae* (*S. pneumoniae*) causes a wide spectrum of human disease, in particular lobar pneumonia. In Europe *S. pneumoniae* is one of the most commonly diagnosed causes of pneumonia (Ieven et al. 2018; Torres et al. 2018). Pneumonia from *S. pneumoniae* often is accompanied by invasive pneumococcal disease (IPD), which is defined as pneumococcal bacterial growth cultured from a normally sterile site such as blood, cerebrospinal fluid or joint fluid. Among potential cofactors, exposure to tobacco smoke increases the risk of pneumonia caused by *S. pneumoniae*, as does ethanol abuse as well as selected comorbidities (Nuorti et al. 2000)*.* However, other factors also appear to be relevant. Growing evidence supports occupational exposure as a clear contributor to risk. Nearly 30 years ago, increased risk of fatal lobar pneumonia was observed among welders in a British register-based study (Coggon et al. 1994). In subsequent studies, increased risk of lobar pneumonia was observed in occupations with possible exposure to metal fumes, such as foundry workers. In those studies, the risk was highest for exposure in the year preceding the disease onset (Palmer et al. 2003, 2009). A prospective cohort study from Sweden in male construction workers found an increased risk of lobar pneumonia and pneumococcal pneumonia among workers occupationally exposed to inorganic dust and metal fumes in the previous year (Torén et al. 2011). There also have been several reports of clusters of pneumococcal pneumonia among welders and shipyard workers with ongoing or recent exposure (Wergeland and Iversen 2001; Ewing et al. 2017; Linkevicius et al. 2019). These investigations focused on clinically or radiographically defined disease, but did not study IPD with pneumonia.

We performed a population-based case–control study of IPD with pneumonia and found that occupational exposures the year preceding pneumonia increased the disease risk (Torén et al. 2020a). The increased risk was evident in relation to occupational exposure to inorganic dust, especially silica dust and fumes, which included metal fumes. Whether long-term, cumulative occupational exposure beyond 1 year increases the risk for pneumonia with IPD remains to be determined.

We hypothesized that increased intensity and duration of exposure additionally increases the risk of IPD with pneumonia, providing a metric of exposure–response further supportive of a causative relationship.

## Materials and methods

### Establishment of study population

The study population was identified by including all IPD cases obtained from a mandatory reporting of communicable diseases in Sweden, SmiNet, a method we have previously reported (Torén et al. 2020a, b). In the current analysis, we have extended the inclusion period from 2006 to 2015 up to 2019. The potentially eligible cases for inclusion (*n* = 11,944) with IPD were those aged 20–80 years with a reported positive culture of *S. pneumoniae* in blood, cerebrospinal fluid, joint fluid, or other normally sterile body fluids. For the present analysis, we excluded those over age 65 (i.e., not of working age) (*n* = 5589 excluded; 6335 remaining). We extracted from the registry the Swedish personal identity number of each case and the date (index date) when the positive sample was obtained. Controls (*n* = 38,076) without IPD were randomly selected from the Swedish National Population Registry. We selected six living controls for each case, matched for gender, age (case year of birth) and region of residency (four urban areas and three rural areas). The rationale for selecting six controls for each case is because that is considered to be an optimal number of controls (Flodin et al. 1981; Axelson 1985). We assigned each control the index date of their corresponding case to define the study observation period. We limited the study to reports from July 1, 2006 through December 31, 2019.

We extracted information from the Swedish national socioeconomic database LISA (Longitudinal integration database for health insurance and labor market studies) on the highest educational level obtained, categorized as: pre-high school (up to 9 years); high school; or university examination. We used this as a surrogate for socioeconomic status (SES). We also extracted income, which we divided in quartiles. From LISA, we also obtained information about the annual occupational history from 2001 until 2019. From the initial population of cases, we excluded those with no reported occupations or with no reported income resulting in a population for analysis of 4689 cases and 31,683 matched controls. Cases and controls were linked with the Swedish National Hospital Discharge Registry and the Swedish Cause of Death Registry to identify the hospitalization or death due to pneumonia (International Classification of Disease (ICD) 10 J10–J18), including any hospital stay that at least included the index date ± 7 days. This resulted in the final study population of 3184 cases with IPD with pneumonia and 16,059 matched controls (Table 1).

**Table 1:** Characteristics of cases with invasive pneumococcal disease (IPD) with pneumonia and matched controls from the general population of Sweden aged 20–65 years

	IPD with pneumonia (*n* = 3184)	Controls (*n* = 16,059)
Males	53.6%, *n* = 1707	54.1%, *n* = 8680
Age, years (SD)	50.7 (11.2)	50.7 (11.0)
Born abroad (outside Sweden)	10.1%, * n* = 323	13.5%, *n* = 2164
Post-high school examination	30.5%, n = 972	37.8%, *n* = 6076
Chronic obstructive pulmonary disease (COPD)^a^	2.6%, n = 83	0.4%, *n* = 57
Bronchial asthma^a^	3.4%, *n* = 107	1.0%, *n* = 158
Diabetes mellitus^a^	9.6%, *n* = 306	4.4%, *n* = 702
Ischemic heart disease^a^	1.9%, *n* = 62	1.5%, *n* = 236
Ethanol abuse	5.2%, *n* = 167	1.8%, *n* = 294
Pneumococcal vaccination	0.2%, *n* = 7	0.1%, *n* = 13
Oral steroids	14.4%, *n* = 457	4.5%, *n* = 716
Immunoactive drugs	5.3%, *n* = 170	1.4%, *n* = 217
Occupational exposure 5 years before index date		
Silica dust		
≤ 5 intensity-years	5.8%, *n* = 186	4.5%, *n* = 714
> 5 intensity-years	0.7%, *n* = 23	0.3%, *n* = 43
Inorganic dust other than silica dust		
≤ 5 intensity-years	23.3%, *n* = 741	20.1%, *n* = 3230
> 5 intensity-years	0.1%, *n* = 2	< 0.1%, *n* = 6
Any inorganic dust		
≤ 5 intensity-years	28.0%, *n* = 890	23.4%, *n* = 3762
> 5 intensity-years	1.0%, *n* = 31	0.7%, *n* = 104
Vapors or gases		
≤ 5 intensity-years	25.0%, *n* = 794	23.4%, *n* = 3762
> 5 intensity-years	2.3%, *n* = 74	0.5%, *n* = 74
Fumes		
≤ 5 intensity-years	23.7%, *n* = 754	21.8%, *n* = 3494
> 5 intensity-years	2.0%, *n* = 62	0.7%, *n* = 104
Organic dust		
≤ 5 intensity-years	9.5%, *n* = 301	10.6%, *n* = 1700
> 5 intensity-years	0.2%, *n* = 6	0.3%, *n* = 50

^a^Hospital-based diagnoses

### Co-morbid conditions

We used the Swedish National Hospital Discharge Registry and the Swedish Prescribed Drug Registry to identify the following comorbid conditions based on ICD 10 coding: Chronic obstructive pulmonary disease (COPD) (ICD 10 J43–J44); asthma (ICD 10 J45); and ischemic heart disease (ICD 10 I20–I25). We defined use of drugs by Anatomical Therapeutic Chemical (ATC) codes. Diabetes mellitus was defined by dispensed diabetes drugs (ATC A10); and ethanol substance abuse disorders as medication dispensed for such disorders (ATC N07B), at any time within 5 years preceding index date. We also extracted information on prescriptions dispensed for oral corticosteroids (ATC code H02A) and immune-modulating medications (ATC code L03–L04) if occurring at any time within 5 years preceding the index date. From the same registry, we also extracted information about pneumococcal vaccinations at any time within 5 years before index date.

### Classification of occupational exposures

Occupation in the 5 years preceding the index date was classified at the four-digit level according to ISCO-88 and ISCO-08 (International Standard Classification of Occupations 1990, 2012) codes. To assess occupational exposures, we used a previously established job-exposure matrix (JEM) for silica dust, inorganic dust other than silica; any inorganic dust; fumes; vapors or gases; and organic dust (Torén et al. 2020a; Lillienberg et al. 2013). The three JEM-defined exposure levels were unexposed, low, or high. Fumes were defined as rising from various processes, such as combustion, fires, welding, or second-hand tobacco smoke. Vapors or gases were defined as substances in aerosol or gas phase. It is of importance to note that an individual could be classified as exposed to multiple categories of agents. We also separately analyzed welders or flame cutters as an occupation with inherent risk of metal fume exposure.

### Statistical methods

We used conditional logistic regression to calculate the ORs of IPD with pneumonia associated with the JEM-defined categories of exposures tested as indicator variables. For each case and control, we calculated a cumulative exposure index, intensity-years, for each exposure by multiplying the duration (maximum 5 years) of each exposure with the level of exposure (0 for unexposed; 1 for low; and 4 for high). We considered that these factors reflected the lognormal distribution of occupational exposures when calculating cumulative exposures. This approach previously has been used in large general-population studies using JEM-based exposure assessments (Sunyer et al. 2005; Lytras et al. 2021). Hence, we analyzed the effect of the exposure in the 5 years preceding the outcome, calculated as the cumulative exposure during those 5 years quantified as intensity-years. We defined ≤ 5 intensity-years as low cumulative exposure and > 5 intensity-years as high cumulative exposure. We also analyzed the risk for welders or flame cutters with 5 years of work, as an indication of metal fume exposure.

We separately performed unadjusted bivariate models (referents matched for age, gender and region of residence, as noted) testing each JEM-based occupational exposure as a predictor of disease but with no other covariates. We further performed additional analyses using models that included the following covariates: comorbid conditions (COPD, asthma, diabetes, ischemic heart disease, oral corticosteroids, and immuno-active drugs in past 5 years, each as indicator variables), educational level, income, having been born abroad, and ethanol abuse. We then performed two multivariable analyses including all the JEM-based categories in the same model along with all the covariates noted previously. One of these models comprised any inorganic dust, but not silica dust and inorganic dust other than silica; the second of these models included silica dust and inorganic dust other than silica as separate predictors, but not any inorganic dust. Full multivariable analyses were repeated in gender-stratified analyses. To further address exposure effects, we carried out a sensitivity analysis of the cumulative exposure restricted to those individuals who had also been exposed in the year before disease onset. The separate analysis of work as a welder or flame cutter as a risk factor for disease included the covariates as noted above.

Confidence intervals (95%) were estimated using exact methods. All analyses were performed using SAS version 9.4 M5 (SAS Inst Inc, Cary, NC, US).

## Results

Among the cases, 28.0% were classified as exposed to any inorganic dust compared to 23.4% among the controls (Table 1). The second most common exposure was vapors or gases for which 25.0% of the cases and 23.4% of the controls were classified as exposed. The pneumococcal vaccination frequency in the previous 5 years was low both among the cases and among the controls, 0.2 and 0.1%, respectively. Diabetes mellitus was a common co-morbidity: 9.6% among the cases and 4.4% among the controls. Use of oral steroids was found among 14.4% of the cases and among 4.5% among the controls. Additional descriptive data are presented in Table 1.

Table 2 summarizes the main results from our bivariate analyses of the different cumulative JEM-categorized occupational exposures associated with IPD with pneumococcal pneumonia. In the separate exposure covariate-adjusted models, high cumulative exposure (> 5 intensity-years) to any inorganic dust (OR 2.17, 95% CI 1.39–3.34), especially silica dust (OR 2.46, 95% CI 1.44–4.18), vapors or gases (OR 2.47, 95% CI 1.83–3.33), as well as high cumulative exposure to fumes (OR 3.08, 95% CI 2.21–4.29), all manifested increased odds for IPD with pneumonia in separate models.

**Table 2:** Logistic multivariable regression models of invasive pneumococcal disease (IPD) with pneumonia in relation to cumulative exposure during 5 years before disease

Cumulative occupational exposure, intensity-years	Invasive pneumococcal disease (IPD) with pneumonia (*N* = 3184)			
	Matched unadjusted models		Adjusted models^a^	
	OR	95% CI	OR	95% CI
Silica dust				
≤ 5 intensity-years	1.37	1.16–1.62	1.27	1.07–1.52
> 5 intensity-years	2.79	1.67–4.67	2.46	1.44–4.18
Inorganic dust other than silica dust				
≤ 5 intensity-years	1.22	1.11–1.34	1.13	1.03–1.25
> 5 intensity-years	N.a	N.a	N.a	N.a
Any inorganic dust				
≤ 5 intensity-years	1.32	1.20–1.44	1.21	1.10–1.34
> 5 intensity-years	2.42	1.57–3.73	2.17	1.39–3.34
Vapors or gases				
≤ 5 intensity-years	1.14	1.04–1.25	1.07	0.98–1.18
> 5 intensity-years	2.59	1.94–3.46	2.47	1.83–3.33
Fumes				
≤ 5 intensity-years	1.14	1.04–1.25	1.08	0.99–1.19
> 5 intensity-years	3.11	2.25–4.28	3.08	2.21–4.29
Organic dust				
≤ 5 intensity-years	0.88	0.77–1.00	0.81	0.71–0.94
> 5 intensity-years	0.63	0.27–1.45	0.60	0.25–1.46

*N.a.* not analyzed due to too few exposed cases (< 5)

^a^Adjusted for educational level, income, chronic obstructive pulmonary disease, asthma, diabetes, ethanol abuse, oral steroids and immune-active drugs; All models are matched for gender, age and place of residency

As the exposure categories can be overlapping, we also performed two multivariable analyses including all the exposures analyzed simultaneously, with separate models for any inorganic dust and for silica dust/inorganic dust other than silica as separate variables, respectively (Table 3). In both models, the increased risk for vapors or gases were attenuated to the null, but the increased risk for both low and high cumulative exposure to any inorganic dust, and silica dust remained, as well as high cumulative exposure to fumes. The cumulative occupational exposure to organic dust was not associated with increased odds, but rather decreased odds among those with low cumulative exposure. Table 4 shows the findings of the multivariable analysis stratified by gender. For silica dust only (and for any inorganic dust), the point estimated odds, where study numbers allowed, were similar among men and women as compared to unstratified data (shown in Table 3). For greater cumulative exposure to fumes, the odds of IPD with pneumonia were attenuated among women.

**Table 3:** Logistic regression models of invasive pneumococcal disease (IPD) with pneumonia in relation to cumulative exposure during 5 years before disease onset

Cumulative occupational exposure, intensity-years	Invasive pneumococcal disease (IPD) with pneumonia (*N* = 2893)			
	Full multivariable model including other occupational exposures including any inorganic dust^a^		Full multivariable model including other occupational exposures including silica dust and inorganic dust other than silica dust	
	OR	95% CI	OR	95% CI
Silica dust				
≤ 5 intensity-years	Not in the model	Not in the model	1.45	1.20–1.76
> 5 intensity-years	Not in the model	Not in the model	2.53	1.49–4.32
Inorganic dust other than silica dust				
≤ 5 intensity-years	Not in the model	Not in the model	1.13	1.00–1.27
> 5 intensity-years	Not in the model	Not in the model	N.a	N.a
Any inorganic dust				
≤ 5 intensity-years	1.23	1.10–1.39	Not in the model	Not in the model
> 5 intensity-years	2.02	1.28–3.19	Not in the model	Not in the model
Vapors or gases				
≤ 5 intensity-years	0.96	0.85–1.07	0.98	0.87–1.11
> 5 intensity-years	1.26	0.82–1.94	1.42	0.93–2.18
Fumes				
≤ 5 intensity-years	1.04	0.93–1.15	1.05	0.94–1.16
> 5 intensity-years	2.31	1.45–3.68	2.24	1.41–3.55
Organic dust				
≤ 5 intensity-years	0.79	0.68–0.91	0.75	0.65–0.87
> 5 intensity-years	0.61	0.25–1.49	0.60	0.24–1.47

*N.a.* not analyzed due to too few exposed cases (< 5)

^a^Matched for gender, age and place of residency and adjusted for educational level, income, chronic obstructive pulmonary disease, asthma, diabetes, ethanol abuse, oral steroids and immune-active drugs

**Table 4:** Logistic multivariable regression models of invasive pneumococcal disease (IPD) with pneumonia among men and women in relation to cumulative exposure during 5 years before disease

Cumulative occupational exposure, intensity-years^a^	Invasive pneumococcal disease (IPD) with pneumonia (*N* = 3184)			
	Full multivariable model including other occupational exposures including any inorganic dust^a^		Full multivariable model including other occupational exposures including silica dust and inorganic dust other than silica dust	
	Males (*n* = 1707)	Females (*n* = 1477)	Males (*n* = 1707)	Females (*n* = 1477)
	OR (95% CI)	OR (95% CI)	OR (95% CI)	OR (95% CI)
Silica dust				
≤ 5 intensity-years	Not in model	Not in model	1.40 (1.13–1.74)	1.71 (1.11–2.62)
> 5 intensity-years	Not in model	Not in model	2.46 (1.44–4.21)	N.a
Inorganic dust other than silica dust				
≤ 5 intensity-years	Not in model	Not in model	1.14 (0.99–1.32)	1.05 (0.83–1.33)
> 5 intensity-years	Not in model	Not in model	N.a	N.a
Any inorganic dust				
≤ 5 intensity-years	1.25 (1.09–1.43)	1.17 (0.92–1.48)	Not in model	Not in model
> 5 intensity-years	2.00 (1.25–3.18)	1.29 (0.06–27.18)	Not in model	Not in model
Vapors or gases				
≤ 5 intensity-years	0.93 (0.81–1.08)	1.00 (0.81–1.24)	0.96 (0.83–1.12)	1.00 (0.81–1.24)
> 5 intensity-years	1.22 (0.78–1.93)	2.17 (0.30–15.78)	1.38 (0.88–2.17)	2.05 (0.40–10.45)
Fumes				
≤ 5 intensity-years	1.02 (0.89–1.17)	1.06 (0.90–1.25)	1.01 (0.88–1.16)	1.10 (0.93–1.30)
> 5 intensity-years	2.40 (1.48–3.88)	0.59 (0.03–10.90)	2.30 (1.43–3.71)	0.71 (0.05–10.57)
Organic dust				
≤ 5 intensity-years	0.77 (0.65–0.90)	0.89 (0.65–1.21)	0.74 (0.63–0.87)	0.82 (0.60–1.14)
> 5 intensity-years	0.40 (0.11–1.43)	1.04 (0.28–3.84)	0.30 (0.11–1.43)	0.97 (0.26–3.60)

*N.a.* not analyzed due to too few exposed cases (< 5)

^a^Adjusted for educational level, income, chronic obstructive pulmonary disease, asthma, diabetes, ethanol abuse, oral steroids and immune-active drugs; all models are matched for gender, age and place of residency

We also analyzed the impact of different 5-year cumulative occupational exposures among those also having been exposed the year before disease (index-year). High cumulative exposure (> 5 intensity-years) to any inorganic dust (OR 1.79, 95% CI 1.08–2.96), silica dust (OR 1.92, 95% CI 1.02–3.58), vapors or gases (OR 2.49, 95% CI 1.79–3.48), as well as high cumulative exposure to fumes (OR 3.29, 95% CI 2.26–4.78) showed increased odds for IPD with pneumonia. The magnitudes of odds ratios were similar from the analysis not requiring an exposed year preceding the disease.

In logistic regression models, the odds for work as welder or flame cutter in the period during 5 years before disease onset was 3.21 (95% CI 1.63–6.32).

## Discussion

This case–control study supports previous evidence that occupational exposure to silica dust or to fumes increases the risk for IPD with pneumonia. Inorganic dust other than silica did not manifest increased risk while cumulative organic dust exposure < 5 exposure years was associated with decreased risk. Further, we found that increasing cumulative exposure to silica dust or fumes during 5 years preceding disease contributes higher risk compared to the lower exposure.

Does cumulative exposure add risk as compared to current exposure only? These results support an affirmative response to that question, at least for inorganic dust. In our previous study we defined current exposure as exposure the year before disease onset, and the odds of IPD with pneumonia for high current exposure to inorganic dust was somewhat lower (OR 1.51, 95% CI 0.93–2.44) compared to the high cumulative exposure in this study (OR 2.02, 95% CI 1.28–3.19) (Torén et al. 2020a, b). Similar pattern is also evident for silica dust where the odds for high current exposure was 1.41 (95% CI 0.78–2.54) in our previous study compared to high cumulative exposure in this study (OR 2.53, 95% CI 1.49–4.32). If cumulative exposures were non-contributory to risk, including it should have attenuated the estimate. In contrast, however, the estimated odds for fumes were quite similar in both studies: 2.71 (95% CI 1.89–3.89) for high current exposure previously compared to 2.24 (95% CI 1.41–3.55) for high cumulative exposure in the current study. Of note, in the pivotal study by Palmer et al. (2003), current exposure to metal fume was associated with an increased risk for pneumonia, whereas exposure to inorganic dust was not.

The mechanisms by which occupational inhalants may increase pneumococcal pneumonia risk are not established, however. There are studies suggesting that the inhalation of metal fumes and silica dust can suppress alveolar macrophages and cause impaired pulmonary clearance of pathogens, resulting in impaired host defense in the respiratory tract (Ghio 2014; di Benedetto et al. 2016). It has also been shown that ultrafine particles present in welding fume increases the adherence of *S. pneumoniae* to the respiratory epithelium (Suri et al. 2016).

We did not observe an increased in relation organic dust exposure, rather a decreased risk. As discussed above, the increased risk associated with inorganic dust and fumes have been linked to metals, especially iron, on the surface of the dust particles (Ghio 2014; di Benedetto et al. 2016). The surface of organic particles appears to be different (Vallyathan et al. 2007; Gustafsson et al. 2018). It is likely that this affect the biological activity of such organic particles.

A major strength of this analysis is it uses national registry data with high, accurate case capture to define the outcome of interest, IPD, in a registry with well-established validity (Ludvigsson et al. 2011). Another study strength is the use of randomly selected population-based controls. Furthermore, we were able to consider a number of key potential confounders using Swedish national health records data.

A central analytic strength of this study is our approach in categorizing occupational exposure. The JEM approach is acknowledged to avoid the recall bias inherent in respondent elicited exposure histories. Nonetheless, the exposure categories we used were partially overlapping, as manifested by the attenuation of the estimated odds ratios for selected exposures, in particular vapors or gases. Further, we were limited to analyzing occupational exposures during 5 years preceding the disease because the registry we used lacked occupational before 2001. The same 5 years’ limitations accounts for our inability to examine pneumococcal vaccination over a 10-year period.

Other study limitations also warrant recognition. Swedish health records could not provide cigarette smoking data, although we could address this in part through including COPD as a covariate. The adjustment for educational achievement, capturing lower educational level, and ethanol abuse, both of which are also linked to smoking status, served to further take that into account (Ali et al. 2009). Nonetheless, residual confounding due to smoking cannot be excluded entirely. We used a broad definition of pneumonia, J10–J18, which includes both viral and bacterial pneumonia. Theoretically, a person with IPD (a prerequisite to case inclusion) might have had a primary viral pneumonia with secondary pneumococcal bacteremia, even though it is doubtful that this was a common source of misclassification.

Contextualizing our study’s strengths and its limitations, its results strongly support the hypothesis that occupational exposure to fumes and inorganic dust, especially silica dust, is associated with increased risk of pneumococcal pneumonia. Previous studies have observed increased pneumonia risk among workers exposed to inorganic dust (Torén et al. 2011, 2020a, b; Koh et al. 2011). This also has historical precedent. The observation that occupational exposure to dust and fume was strongly linked to pneumococcal pneumonia was repeatedly reported by German researchers in 1930s (Gundel and Heine 1938). This link was rediscovered in the 1990s and thereafter (Coggon et al. 1994; Palmer et al. 2003, 2009).

Our study, together with a body of supporting literature, leads to the conclusion that pneumococcal pneumonia should be regarded as an occupational disease in workers with high cumulative exposure to silica dust and fumes. The basic prevention of this form of occupational illness should be the reduction of workplace exposure to dust and fumes, a risk that continues to be poorly controlled among certain occupations, for example welders and others. We also observed threefold increased odds of IPD with pneumonia among welders and flame cutters, consistent with a particular role of metal fumes, which were also represented in the more heterogeneous JEM category fumes. It may also be advisable to provide additional protection of persons with such exposure through pneumococcal vaccination (Coggon et al. 2015). Critically, for these exposures, where the odds exceed two even after adjustments for other factors, in an individual person with both exposure and pneumonia, the disease is more likely than not to be work-related, given a personal attributable risk > 50%. Thus, the most effect prevention of future disease may be recognition and industrial compensation for the cases that continue to accrue.

## Declarations

### Data availability

The study is based on national registers, which needs a Swedish ethical application to be analyzed. The lead author (KT) affirms that the manuscript is an honest, accurate, and transparent account of the study being reported; that no important aspects of the study have been omitted; and that any discrepancies from the study as planned have been explained.
